# Geographic variation in incubation behavior of a widely distributed passerine bird

**DOI:** 10.1371/journal.pone.0219907

**Published:** 2019-08-14

**Authors:** Vanya G. Rohwer, James R. Purcell

**Affiliations:** Cornell University Museum of Vertebrates, Ithaca, NY; Universidad de la Republica Uruguay, URUGUAY

## Abstract

Incubating birds must trade-off leaving the nest to forage with staying on the nest to maintain optimal temperatures for developing embryos. This trade-off is expressed through incubation behavior, which can be heavily influenced by climate, food availability, attentiveness of their mates, and nest predation risk. Comparative studies across species have shown that incubation behavior varies across latitude, but few studies have explored how incubation behavior varies across sites within species. We might expect incubation behavior to be flexible and respond to local environmental challenges; alternatively, behavior may be relatively fixed and vary little across a species’ range. We explored four incubation behaviors (male feeding rate, female off-bout duration, female off-bout frequency, and the proportion of time incubating females spent on the nest) in a widespread songbird, the yellow warbler (*Setophaga petechia*), breeding at a temperate and subarctic site. As temperatures warmed at both sites, males fed females less often, and as male feeding rates decreased, off-bout durations and frequencies increased causing the proportion of time on the nest to decrease. While incubation behaviors changed in similar ways between sites, off-bout durations shortened with increasing male feeding rates most strongly at the temperate site. Overall, these results show flexibility in incubation behaviors in response to different environmental cues, which likely minimize costs associated with provisioning incubating parents and maintaining warm nest temperatures, and suggests that male feeding may be especially important for breeding in cold regions.

## Introduction

The incubation period is a unique stage in the lives of adult birds and developing embryos. Once eggs are exposed to the warm temperatures of an incubating parent, embryos must be maintained within a narrow range of temperatures for optimal development [1–3]. Temperatures that fall below 24–26°C, will slow development and can negatively affect survival of young [3,4], while temperatures that rise above 40.5°C can be lethal to developing embryos [1,3]. For incubating parents, maintaining these temperatures can be challenging when ambient temperatures differ from those optimal for embryo development [5]. Cold ambient temperatures challenge adults to provide consistent, warm incubation temperatures [6], and also force them to feed more often to meet their increased metabolic demands [7,8]. By contrast, hot ambient temperatures challenge adults to prevent eggs from overheating or losing too much water, but also require adults to sustain prolonged exposure to heat [9,10]. Overcoming these challenges often requires parents to change their behaviors to balance risks associate with survival of their eggs and survival of themselves. To much activity around the nest site can cue predators and increase the risk of nest predation [11–14], while inconsistent incubation temperatures that deviate from the optimal thermal range can result in extended incubation periods [3], compromised offspring quality [4], or even death of the developing embryo [1].

How parents overcome these challenges depends on how closely males and females share costs of incubation. At one extreme, only a single parent incubates and at the other extreme, both parents share incubation costs equally. Challenging breeding conditions, such as those posed by high elevation, high latitude, or extremely hot and arid sites, are thought to favor increased cooperation between males and females, as both parents share fitness interests in successful incubation [15]. For incubating birds, increased cooperation should be observed through (i) increased male feeding rates of incubating females, or (ii) more equally shared incubation durations between parents [16]. However, when fitness interests are not equally shared between males and females, or if favorable environmental conditions do not requires close cooperation for successful incubation, we might expect the contributions of parents to differ during incubation. Specifically, if the fitness gains of parents are maximized by activities other than successful incubation (e.g., males that seek extra pair copulations) then costs of incubation should be unequal among parents [17,18].

Comparative and experimental studies have revealed patterns in male and female incubation behaviors that broadly follow patterns predicted by environmental challenges during incubation. Species with high risk of nest predation have evolved both long on- and off-bouts from the nest, and reduced male feeding to incubating females, all of which minimize activity around the nest site and the risk of cueing predators [11,12,14,19]. As temperatures drop below or rise above levels optimal for embryo development, parents will share costs of incubation more equally (usually by males feeding incubating females more frequently allowing females to remain on the nest for longer periods of time or, in species with biparental incubation, males will spend more time incubating/attending eggs), providing more stable environments for developing embryos [7,16,20,21]. Ambient temperatures can also influence nest morphology, placement, and food availability, all of which can affect incubation behavior. Species and populations often construct nest morphologies that match local environmental conditions [22,23], balancing the energetic needs of incubating parents with the likelihood of re-nesting attempts [24–27]. Finally, for species that feed primarily on insects, extreme temperatures may reduce insect activity [28], forcing birds to search for food longer and result in extended or more frequent female off-bouts, or less frequent male feedings.

Given that climate, predation, and food availability affect incubation behavior across species [12,14], variation in these same factors across the breeding range of a single species should influence incubation behaviors between populations. However, data on geographic variation in incubation behaviors within species is scarce (incubation behaviors [20,21,29]; nestling care [30–33]), despite wide variation in climate and predation risks across breeding sites [34,35]. Not only should climate and predation pressure differ across the breeding range [34,35], but so too should life-history strategies [36–38], all of which may influence incubation behaviors. Life-history theory predicts that populations breeding at high latitude sites should invest more in current reproduction at the costs of future reproductive opportunities, especially if the likelihood of subsequent breeding attempts is small [37]. For incubation behaviors, these differential investments should be expressed through greater nest attentiveness.

While these predictions follow from observations made across species, anecdotal information from within species suggests that incubation behaviors may show less flexibility than previously thought. On the one hand, incubation behaviors should be flexible and respond to changing environmental conditions experienced by the parents [20,21]. For example, several species increase nest attentiveness (the percentage of time females spend on the nest) when provided with supplemental food, supporting the idea that incubation behaviors respond to changes in food abundance (studies summarized in [39]); however, these increases in nest attentiveness are typically small, suggesting food shortages do not drive broad patterns in female incubation behavior. On the other hand, incubation behaviors within species may represent evolved strategies that are relatively constrained in their ability to change in response to different challenges during incubation. For example, when crested mynas (*Acridotheres cristatellus*), which are native to the tropical Indochinese region, were introduced to British Columbia, their incubation behaviors remained similar to those observed in their native range despite significantly lower ambient temperatures in Canada compared to Indochina [40].

In this study, we explore variation in incubation behaviors between two populations of yellow warblers, *Setophaga petechia*, breeding at a subarctic and temperate site. The yellow warbler is a widespread songbird that breeds throughout much of North America, exposing them to diverse climatic and biotic conditions that may favor different incubation behaviors. Yellow warblers build cup-shaped nests and only females incubate, but males will feed females during incubation [41]. Male yellow warblers vary in the amount of rufous streaking on their breast and belly and this plumage variation is thought to correlate with parental investment [42,43], which could influence male feeding rates during incubation. Yellow warblers also show striking geographic variation in nest morphologies with subarctic-breeding females constructing thicker, better-insulated nests compared to temperate-breeding females [44,45]. Previous experiments that transplanted nests between subarctic and temperate sites, however, found no differences in incubation behavior as a result of different nest morphologies, at least at the subarctic site [35]. This suggests that that the effect of nest morphology on incubation behavior is small and only expressed under more severe temperature or predation regimes, or that incubation behavior varies little with changing nest microclimates.

The subarctic site presents yellow warblers with colder ambient temperatures, higher winds, lower nest predation rates, and fewer nest ectoparasites compared to the temperate site [35]. Given the different challenges posed by subarctic and temperate sites, we predicted that male yellow warblers would feed incubating females more frequently and that females would have shorter, more frequent off-bouts, allowing for increased nest attentiveness at the subarctic site compared to the temperate site [7,46].

## Methods

### Data collection

We filmed 16 pairs of yellow warblers at a temperate site near Westport, Ontario (N: 44° 30’ W: 76° 19’ elev 125m) and 30 pairs at a subarctic site near Churchill, Manitoba, (N: 58° 40’ W: 94° 25’, elev 20m) from late May to early July in the summers of 2008, 2009, 2011, and 2012. All nests were videotaped during the early stages of incubation (between days 2 and 4) using a Sony Handycam DCR-SR85 video camera. For this study, each pair was filmed once. Cameras were placed on a tripod approximately 6–10 m from the nest, and each nest was filmed for 5–7 hours, beginning around 06:00 at the temperate site and 04:00 at the subarctic site; in both locations filming started in the early morning before sunrise. For all filmed nests, we recorded nest height and clutch size.

We measured four aspects of incubation behavior: male feeding rate (the average number of times males fed incubating females per hour), off-bout frequency (average number of times the female left the nest per hour), off-bout duration (average time the female spent off the nest during each off-bout), and the proportion of time females spent on the nest for the duration of filming. We calculated all behaviors as rates or proportions to control for videos of different lengths at each nest. To reduce the effects of possible disturbance from camera set-up on male and female incubation behaviors, all male feedings and female departures that occurred within 10 minutes of set-up were excluded from analyses.

In addition to behavioral data gathered from videos, we assembled hourly temperature and precipitation data for both study sites using Environment Canada’s online database (Environment Canada http://climate.weather.gc.ca/historical_data/search_historic_data_e.html), which has weather stations within 15 km of each study site. For each videotaped pair, we recorded the minimum temperature during which the video was taken, as well as presence or absence of precipitation.

### Assessing male plumage

We assessed the amount of reddish streaking found in the breast and flanks of male yellow warblers because male investment is thought to vary with this plumage character during breeding. Heavily streaked males achieve more extra pair matings [43], occupy higher quality habitats, and are thought to feed incubating females less than lightly streaked males [42], all of which could influence a male’s behavior during incubation. We quantified variation in male plumage from video footage by assigning individuals a score of 1 through 10, where 1 represents sparse streaking and 10 represents heavy streaking. We incorporated plumage scores from as many males as we could confidently assess plumage from video footage (subarctic: *n* = 15; temperate: *n* = 15); males excluded from this analysis could not be scored for plumage characters through video footage. We used museum specimens of male yellow warblers as a reference for the streaking index (see Figure A in S1 File).

### Data analysis

We tested for differences in all four incubation behaviors (male mate feeding, proportion of time on the nest, off-bout frequency, and off-bout duration) between subarctic and temperate sites using linear models in R [47]. For the analysis of male behavior, the response variable was male feeding rate and the predictor variables were location, minimum temperature throughout the video period, presence or absence of precipitation, clutch size, nest height, male plumage score, and an interaction term between location and minimum temperature. Because we lacked plumage data for all males, we ran this analysis using all individuals that had complete data then dropped male plumage score, as this variable was not significant, to increase sample sizes. For analyses of female incubation behaviors, we ran three different models each with a different dependent variable (off-bout frequency, off-bout duration, and proportion of time on the nest), but all models had the same predictor variables of location, male feeding rate, minimum temperature throughout the video period, presence or absence of precipitation, clutch size, nest height, and an interaction term between location and male feeding rate. For female off-bout frequency and duration we log10 transformed these data so that they better fit the assumptions of our analyses, but we present untransformed data in figures.

We used Akaike’s Information Criterion corrected for small samples sizes (AICc), and selected the top performing models using the *dredge* function in package MuMIn [48]. For all analyses, we checked the assumptions and fit of models following Zuur et al. [49], by testing for normality of model residuals using Shapiro-Wilks tests, plotting model residuals on predictor variables to confirm that no patterns between residuals and predictor variables emerged, and testing for differences in the variance between categorical variables using Bartlett’s tests. For all analyses, we present summaries of top performing models (those that were within 2 AICc values of the best performing model) and parameter estimates averaged across all top performing models, weighted by model performance (Tables 1 and 2).

**Table 1:** Top performing models (ΔAICc < 2) for factors influencing four incubation behaviors across a subarctic (*n* = 30) and temperate (*n* = 16) breeding site.

Factors influencing male feeding rates					
	df	Log likelihood	AICc	ΔAICc	Weight
Ambient temperature	3	-127.35	261.28	0	0.71
Ambient temperature + location	4	-127.04	263.05	1.77	0.29
Factors influencing off-bout frequency per hour					
	df	Log likelihood	AICc	ΔAICc	Weight
Male feeding rate + location	4	22.67	-36.37	0	0.29
Male feeding rate	3	21.39	-36.21	0.15	0.27
Male feeding rate + nest height	4	22.30	-35.63	0.73	0.20
Male feeding rate + location + ambient temperature	5	23.12	-34.74	1.63	0.13
Male feeding rate + location + nest height	5	23.06	-34.61	1.75	0.12
Factors influencing off-bout duration					
	df	Log likelihood	AICc	ΔAICc	Weight
Male feeding rate + location + nest height + male feeding rate:location	6	29.14	-44.12	0	0.27
Male feeding rate + location + male feeding rate:location	5	27.14	-43.64	0.48	0.21
Male feeding rate + nest height	4	26.26	-43.55	0.57	0.20
Male feeding rate + location + clutch size + nest height + male feeding rate:location	7	29.69	-42.44	1.68	0.12
Male feeding rate + location + nest height	5	26.83	-42.17	1.95	0.10
Male feeding rate + location + precipitation	5	26.82	-42.15	1.97	0.10
Factors influencing the proportion of time on the nest					
	df	Log likelihood	AICc	ΔAICc	Weight
Male feeding rate	3	86.65	-166.74	0	0.47
Male feeding rate + precipitation	4	87.52	-166.07	0.67	0.34
Male feeding rate + nest height	4	86.94	-164.91	1.83	0.19

For analysis of male feeding rates, our model included predictor variables: location, minimum ambient temperature throughout the video period, presence or absence of precipitation, clutch size, nest height, male plumage score, and an interaction between location and minimum ambient temperature. For analyses of female incubation behaviors, all models included predictor variables: location, male feeding rates, minimum ambient temperature throughout the video period, presence or absence of precipitation, clutch size, nest height, and an interaction between location and male feeding rates.

**Table 2:** Parameter estimates averaged from all top performing models in Table 1 for each corresponding incubation behavior; bold face indicates variables that were statistically significant for each analysis.

Male feeding rates				
	Estimate	SE	z	p
**Ambient temperature**	**-0.570**	**0.142**	**7.827**	**<0.0001**
Location (temperate)	-1.069	1.387	0.749	0.454
Off-bout frequency				
	Estimate	SE	z	p
**Male feeding rate**	**-0.018**	**0.005**	**3.292**	**0.0009**
Location (temperate)	-0.080	0.053	1.457	0.145
Ambient temperature	0.006	0.007	0.882	0.377
Nest height	-0.053	0.048	1.081	0.279
Off-bout duration				
	Estimate	SE	z	p
**Male feeding rate**	**-0.021**	**0.005**	**3.624**	**0.0003**
Location (temperate)	0.171	0.090	1.851	0.064
**Male feeding rate:location**	**-0.031**	**0.015**	**2.018**	**0.043**
Clutch size	0.038	0.039	0.960	0.337
Nest height	0.079	0.043	1.770	0.077
Precipitation (present)	-0.065	0.064	0.988	0.323
Proportion time on the nest				
	Estimate	SE	z	p
**Male feeding rate**	**0.008**	**0.001**	**6.697**	**<0.0001**
Precipitation (present)	0.021	0.017	1.248	0.212
Nest height	-0.008	0.011	0.715	0.474

### Ethical statement

Videotaping females during incubation is noninvasive and was approved by the Queen’s University Animal Care and Use Committee (protocol number: Martin-2008-025-R2) and with permission from the Canadian Wildlife Service (permit number: CA 0223). We were careful not to disturb vegetation around the nest during camera placement and removal, and most females remained on the nest during these events.

## Results

**Fig 1:**
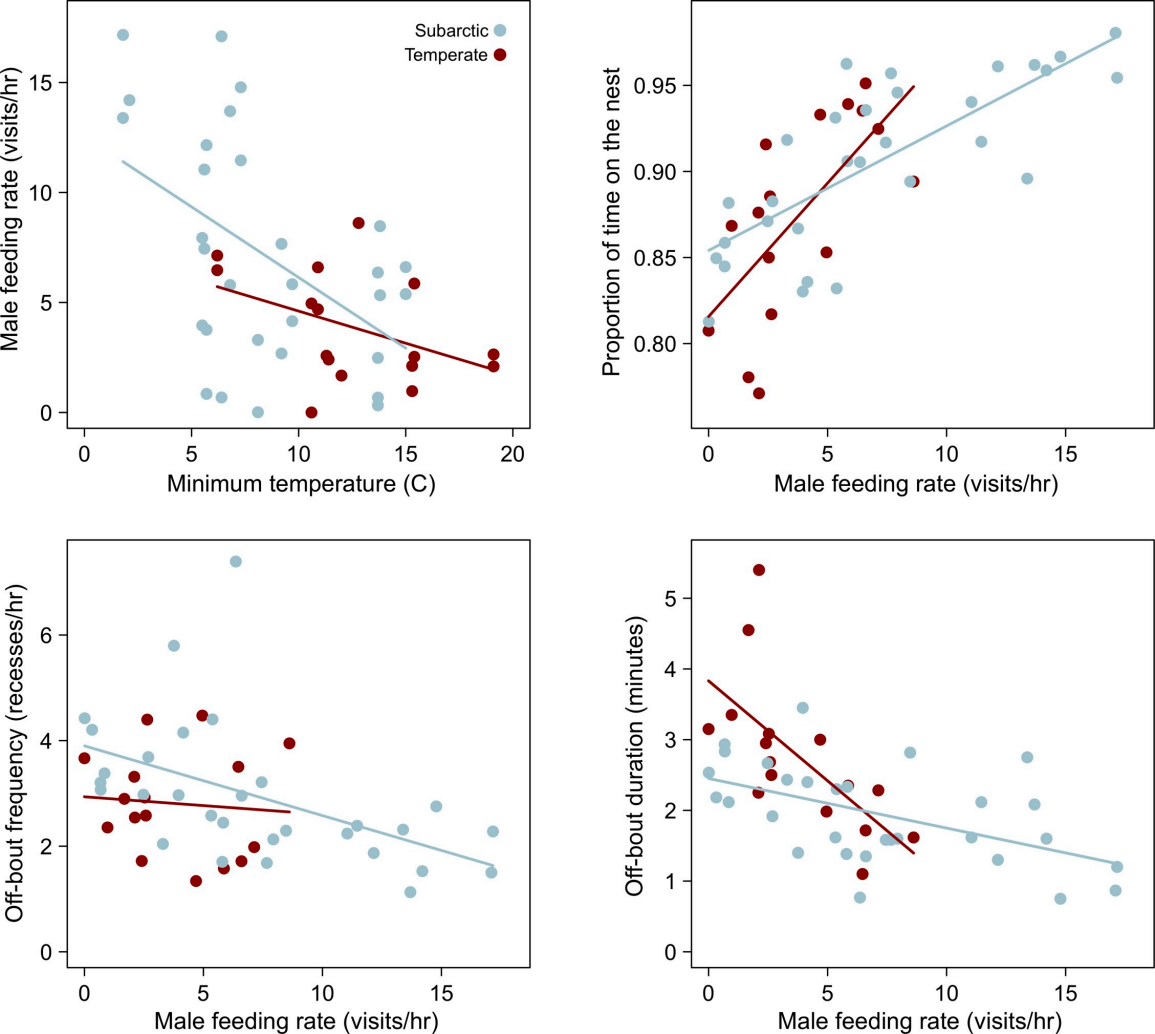
Yellow warbler incubation behavior at a subarctic and temperate site. Variation in incubation behavior when correlated with significant predictor variables of minimum temperature and male feeding rate. Slopes did not differ between subarctic and temperate sites for three behaviors (male feeding rate, proportion of time on the nest, and off-bout frequency) but are plotted to illustrate patterns with minimum temperature and male feeding rates. Slopes between subarctic and temperate females were significantly different for off-bout duration (*p* = 0.04). Sample sizes for all behaviors were: subarctic: *n* = 30; temperate *n* = 16.

Yellow warblers showed no significant differences in incubation behaviors between breeding sites (Fig 1), after controlling for ambient temperature and male feeding rates. As temperatures decreased, male feeding rates increased, and as male feeding rates increased, female off-bout frequency and duration decreased while the proportion of time females spend on the nest increased (Fig 1). Variation in male feeding rates was best explained by variation in minimum temperature (*p* = 0.0037) (Tables 1 and 2). For female incubation behaviors, male feeding rate was the only significant predictor variable for off-bout frequency, (*p* = 0.013) and the proportion of time on the nest (*p* < 0.0001), after controlling for minimum temperature. Finally, both male feeding rate (*p =* 0.0003) and an interaction between location and male feeding rate (*p* = 0.044) were significant predictors for off-bout durations of incubating females (Tables 1 and 2); it is important to note that unequal variation in male feeding rates between sites (i.e., 0–9 visits/hr at the temperate site versus 0–17 visits/hr at the subarctic site) may be creating or masking site-specific effects that male feeding rates have on female incubation behaviors.

## Discussion

Species that breed across a broad geographic range face different challenges to successful reproduction in different locations. We examined four incubation behaviors in yellow warblers between two sites, separated by over 14 degrees of latitude, which present breeding warblers with different climatic and predation pressures [35]. Despite difference in environmental challenges between these sites, yellow warblers did not vary in their incubation behaviors after controlling for differences in ambient temperature and male feeding rates (Fig 1). Males fed females less frequently as temperatures warmed, and as male feeding rates decreased, females took longer off-bouts and spent less time on the nest (Fig 1). These patterns suggest that incubation behaviors between sites are flexible and respond to local conditions, and that high rates of male feeding may be especially important when breeding in cold environments.

Studies of incubation behavior in passerines have revealed two broad patterns in relation to predation and temperature: when predation rates are high, males and females reduce activity around the nest [11,14,19], and when temperatures are low, females spend more time on the nest and males feed females more frequently [7,14,50]. Yellow warbler incubation behaviors show patterns consistent to those of comparative studies. At the subarctic site, where predation rates are lower and ambient temperatures cooler [35], males showed some of the highest feeding rates of incubating females, and females generally took short off-bouts regardless of male feeding rates, allowing females to spend a higher proportion of time on the nest (Fig 1). At the temperate site, where predation rates are higher and temperatures warmer [35], females showed wide variation in the duration of off-bouts, but relatively little variation in off-bout frequency and male feeding rates, suggesting that activity around the nest may be constrained by higher predation risk [12,35]. Thus, when ambient temperatures are more severe than the risk of nest predation, incubation behaviors that allow females to spend more time on the nest should change most, whereas when predation risk is more severe than ambient temperatures, incubation behaviors should shift to reduce activity around the nest site [7,12].

The subarctic site sits at the northern limit of the yellow warbler breeding range, where the contribution of male feeding likely plays a more important role to successful incubation compared to temperate sites. When temperatures approached freezing, males at the subarctic site showed some of the highest feeding rates (17 trips/hour), which corresponded with the lowest off-bout frequency and shortest off-bout durations, allowing females to remain on the nest during these cold temperatures. These male feeding rates are higher than those of other species [11,14,50] and those of temperate breeding yellow warblers (although these males were not exposed to similar cold temperatures), suggesting that subarctic breeding females may be especially reliant on male provisioning during cold weather. As temperatures drop, the costs of female off-bouts increases because exposed eggs cool more quickly, slowing or even halting development, which could effect nestling phenotype and future survival [3,4,51]. Thus, the role of male provisioning may be especially important during cold severe weather by allowing females to remain on the nest, facilitating successful breeding at the northern limit of the range.

Subarctic females must maintain their energy balance while incubating during cold spells. Females at the subarctic site took shorter off-bouts for similar male feeding rates as temperate females, but similar off-bout frequencies, suggesting that (i) increased male provisioning compensated for less time spent away from the nest, (ii) females accumulated an energy deficit during incubation, or (iii) food resources were more abundant or higher quality and females needed less time to forage. Given the high rates of male feeding during cold temperatures, we suspect that males increase their provisioning rates to allow increased nest attentiveness by females, similar to other warbler species during nestling brooding [33]. Alternatively, if females accrue an energy deficit, differences in life-history strategies between subarctic and temperate females may explain differences in off-bout durations between sites. Birds breeding at high latitudes tend to have lower adult survival and invest more heavily in their current reproduction at the costs of future reproductive opportunities [37], thus subarctic breeding females may invest more heavily in incubation at the expense of their own energetic needs.

Male feeding rates showed remarkable variation in the subarctic. Some males made as many as 17 trips/hour to feed incubating females while other males made none (Fig 1). In fact, some incubating females stopped accepting food delivered by males, presumably because they were satiated. We found no relationship between male streakiness and feeding rates suggesting that plumage characters associated with alternative reproductive strategies were not responsible for variation in male feeding rates [42,43], despite male plumage scores ranging from 4–9 in our analyses (see Figure B in S1 File). If alternative reproductive strategies drove the observed variation in male feeding rates, this variation might be expressed when temperatures are warmest and males most able to seek extra pair matings. However, variation in male feeding rates at the subarctic was most pronounced at relatively cold temperatures (~5°C), suggesting that low temperatures express individual variation in either territory or individual quality that warmer temperatures did not reveal.

Differences in incubation behavior result in temperate and subarctic yellow warblers spending different amounts of time on and off the nest, which should affect incubation duration. However, we found no difference in incubation durations between study sites (Rohwer et al. unpublished data; but see Briskie [44] who reports an average of 0.4 day longer incubation period at a subarctic site compared to a temperate site). One possible explanation is differences in daylight hours between sites. At the subarctic site the sun is up for 18.42 hours on June 21^st^ (the summer solstice) while at the temperate site, the sun is up for 15.52 hours on the solstice, almost 3 hours less each day (these differences become more pronounced because many temperate breeding warblers start incubating nearly a month sooner than the summer solstice). Thus, temperate females have nearly 3 more night-time hours to provide consistent incubation temperatures compared to subarctic females. Multiplying the average proportion of time a female spends on the nest with the daylight hours specific to her breeding locality (subarctic: 0.90 prop time on nest * 18.42 sun-up hours = 16.58 hours on the nest; temperate: 0.87 prop time on nest * 15.52 sun-up hours = 13.50 hours on the nest), shows that differences in time spent on the nest during sun-up hours (subarctic: 16.58; temperate: 13.50; difference: 3.08 hours) are remarkably similar to the difference in daylight hours between locations (2.90 hours). Thus, while temperate females may spend more time away from their nest during daylight hours, they make up for this during longer night-time incubation sessions and have incubation periods nearly identical to subarctic breeding females.

## Conclusions

Yellow warblers breeding in a subarctic and temperate site did not differ in their incubation behaviors after accounting for differences in ambient temperature and male feeding rates between sites. The variation in environmental conditions and subsequent changes in male feeding rates during cold temperatures suggests that challenging environmental conditions favor increased cooperation among males and females. Increased cooperation is largely driven by more frequent male feeding visits, apparently a facultative response to environmental cues (e.g., temperature, food availability, nest predators), and likely not an evolved strategy specific to populations, a result that mirrors findings in other species: [16,20]. High male feeding rates appear most important to successful reproduction at the subarctic site, where females must remain on the nest during bouts of cold severe weather. The overall flexibility in yellow warbler incubation behaviors combined with geographic differences in their nest morphologies [44,45], suggests that a suite of breeding behaviors respond to environmental conditions and allow populations to cope with diverse ecological challenges during the breeding season.

## Supporting information

S1 File **Figure A.** Variation in the extent of rufous streaking in the belly and flanks of male yellow warblers, which is thought to co-vary with reproductive strategies and male feeding rates. In our scoring of male plumage, individuals with little streaking (left) received low scores (1), while individuals with heavy streaking (right) received high scores (10). **Figure B.** We found no relationship between male feeding rates and male plumage score, using all males for which we could score plumage characters from video footage (subarctic: *n* = 15; temperate: *n* = 15).
(DOCX)

S1 Data This file includes all data used in our analyses. (TXT)
