# Supplementary figures and images for: Geographic variation in incubation behavior of a widely distributed passerine bird
Source: PLoS One. 2019 Aug 14;14(8):e0219907. doi: 10.1371/journal.pone.0219907 (PMC6693686; doi:10.1371/journal.pone.0219907)

**Figure A.**


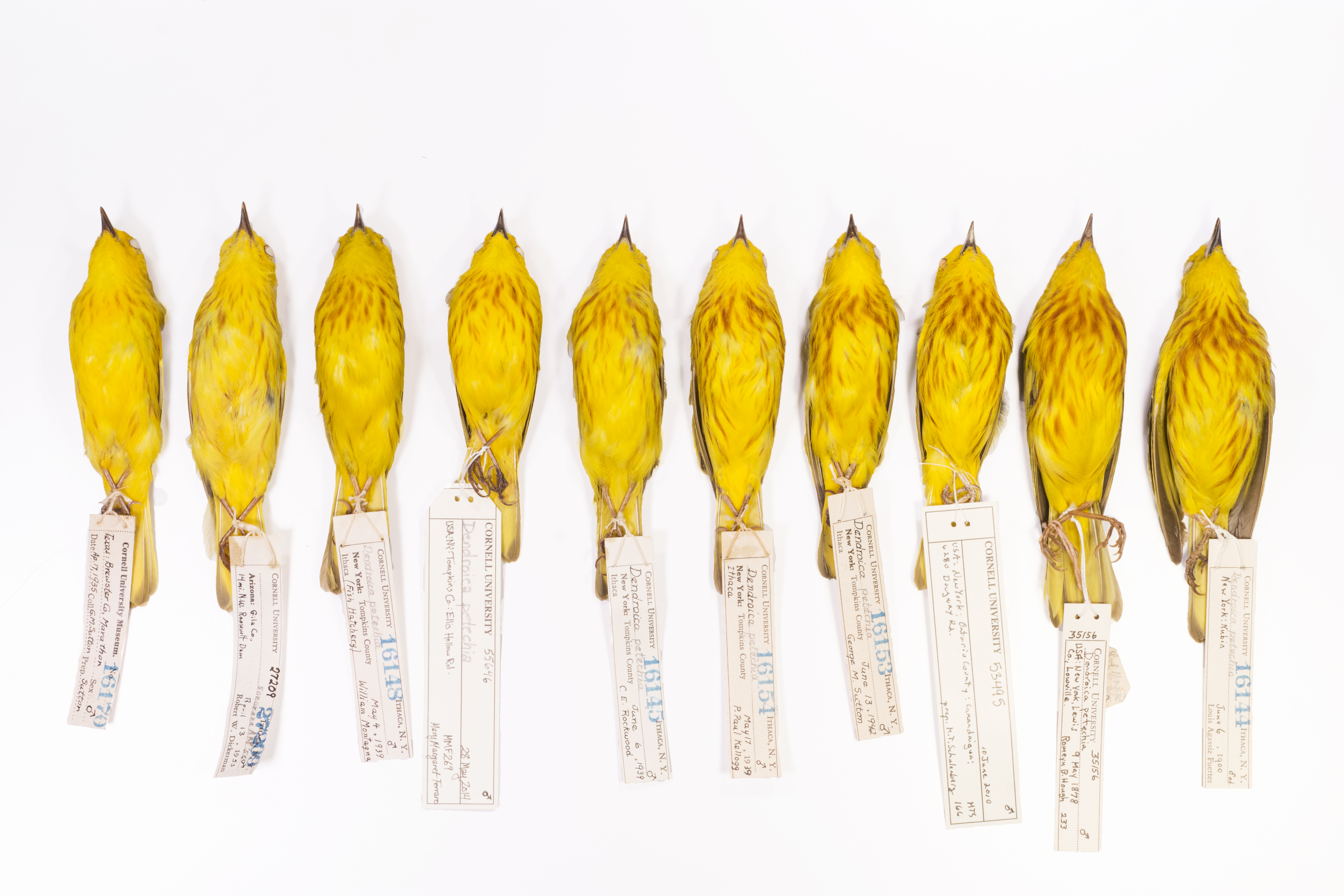


**Figure B.**

Supplement: S1 File — Figure A. Variation in the extent of rufous streaking in the belly and flanks of male yellow warblers, which is thought to co-vary with reproductive strategies and male feeding rates. In our scoring of male plumage, individuals with little streaking (left) received low scores (1), while individuals with heavy streaking (right) received high scores (10). Figure B. We found no relationship between male feeding rates and male plumage score, using all males for which we could score plumage characters from video footage (subarctic: n = 15; temperate: n = 15). (DOCX) [file pone.0219907.s001.docx]
